# Enhanced BDNF Actions Following Acute Hypoxia Facilitate HIF-1α-Dependent Upregulation of Cav3-T-Type Ca^2+^ Channels in Rat Cardiomyocytes

**DOI:** 10.3390/membranes11070470

**Published:** 2021-06-25

**Authors:** Masaki Morishima, Takafumi Fujita, Satoshi Osagawa, Hiroshi Kubota, Katsushige Ono

**Affiliations:** 1 Department of Food and Nutrition, Kindai University Faculty of Agriculture, Nara 631-8505, Japan; mmoris@nara.kindai.ac.jp; 2 Department of Pathophysiology, Oita University School of Medicine, Oita 879-5593, Japan; cardio2000xt@gmail.com (T.F.); r2d20012001@gmail.com (S.O.); heartland20012001@gmail.com (H.K.)

**Keywords:** BDNF, TrkB, HIF-1α, Cav3.1, Cav3.2, T-type Ca^2+^ channel, hypoxia

## Abstract

Brain-derived neurotrophic factor (BDNF) has recently been recognized as a cardiovascular regulator particularly in the diseased condition, including coronary artery disease, heart failure, cardiomyopathy, and hypertension. Here, we investigate the role of BDNF on the T-type Ca^2+^ channel, Cav3.1 and Cav3.2, in rat neonatal cardiomyocytes exposed to normoxia (21% O_2_) and acute hypoxia (1% O_2_) in vitro for up to 3 h. The exposure of cardiomyocytes to hypoxia (1 h, 3 h) caused a significant upregulation of the mRNAs for hypoxia-inducible factor 1α (*Hif1α*), *Cav3.1*, *Cav3.2* and *Bdnf*, but not tropomyosin-related kinase receptor B (*TrkB*). The upregulation of *Cav3.1* and *Cav3.2* caused by hypoxia was completely halted by small interfering RNA (siRNA) targeting *Hif1a* (*Hif1a*-siRNA) or *Bdnf* (*Bdnf*-siRNA). Immunocytochemical staining data revealed a distinct upregulation of Cav3.1- and Cav3.2-proteins caused by hypoxia in cardiomyocytes, which was markedly suppressed by *Bdnf*-siRNA. These results unveiled a novel regulatory action of BDNF on the T-type Ca^2+^ channels expression through the HIF-1α-dependent pathway in cardiomyocytes.

## 1. Introduction

Myocardial infarction is a major cause of death in many countries [1]. The effect of regional myocardial ischemia and hypoxia on myocardial function has widely been studied in patients and animals to understand the mechanism of structural and electrical remodeling of the heart after myocardial infarction, because it eventually causes progressive myocardial dysfunction or heart failure. Electrophysiological changes in cardiomyocytes caused by ion channel remodeling may play a key role in arrhythmia generation in the post-myocardial infarction heart. Arrhythmogenic remodeling involves alterations in many ion channel species including the K^+^ channels, the Na^+^ channels and the Ca^2+^ channels [2,3]. Two types of Ca^2+^ channels are expressed in cardiac myocytes: the L-type Ca^2+^ channels and the T-type Ca^2+^ channels. The L-type Ca^2+^ channels are abundantly expressed and play crucial roles in excitation–contraction coupling, pacemaker depolarization, and electrical conduction [4,5]. On the other hand, the T-type Ca^2+^ channels are expressed in embryonic and neonatal myocytes but are scarcely expressed in adult ventricular myocyte [3,6]. The strongest evidence of their function is that T-type Ca^2+^ channels participate in pacemaking in the sinoatrial node [3,7].

The re-expression of the T-type Ca^2+^ current has been observed in the diseased condition of the heart including post-myocardial infarction [2]. Nevertheless, studies of the action of hypoxic insult on the expression of the T-type Ca^2+^ channels are controversial and not convincing [8,9,10,11,12,13].

Brain-derived neurotrophic factor (BDNF) was originally identified in the brain as a member of the neurotrophin family [14]. BDNF specifically binds to the tropomyosin-related kinase receptor B (TrkB) and activates multiple intracellular signaling pathways in many different types of cells [14]. Recent emerging evidence suggests that BDNF is also required for the physiological and pathophysiological function in the heart [15,16]. Of note, several studies demonstrate that cardiac hypoxia/ischemia triggers the elevation of BDNF production in cardiomyocytes, suggesting variable roles of BDNF including for angiogenesis, contractility, anti-apoptosis, anti-oxidative, reducing infarction area, and mitochondrial biogenesis [17,18,19]. However, it remains unclear whether BDNF is involved in the ion channel remodeling after hypoxic insult such as in myocardial infarction. Discrepancies of hypoxic insults on the T-type Ca^2+^ channel expression and the growing interests to the action of BDNF motivated us to investigate the impact of hypoxic insult in combined with BDNF on the Cav3.1- and Cav3.2-T-type Ca^2+^ channels expression in cardiomyocytes.

In the present study, we unveiled a novel action of BDNF as a modulator of Cav3.1- and Cav3.2-T-type Ca^2+^ channels in the hypoxic conditions of the heart.

## 2. Results

### 2.1. Acute Hypoxia Upregulates Cav3.1, Cav3.2 and Bdnf

**Figure 1:**
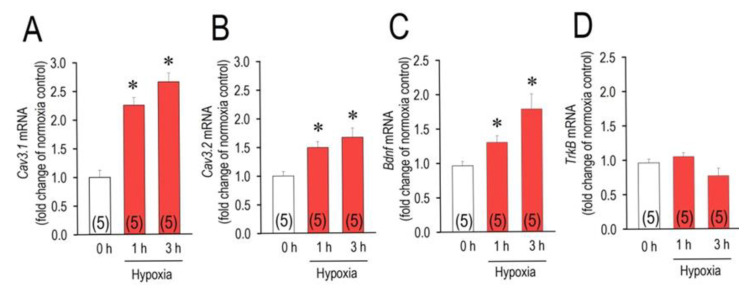
Changes of the levels of *Cav3.1*-, *Cav3.2*-, *Bdnf*-, and *TrkB*-mRNA expression in cardiomyocytes exposed to hypoxia. Rat neonatal cardiomyocytes were cultured in the normoxic conditions (21% O_2_) for 3 h or hypoxic conditions (1% O_2_) for 1 h or 3 h. Expression of each mRNA (**A**–**D**) was assessed by real-time PCR. Amount of each mRNA was normalized to those in normoxia (0 h) as assigned as 1.0. Data were expressed as mean ± SE. (n = 5). * *p* < 0.05, compared with those in the normoxic control group (3 h). Numbers of experiments are given in parentheses.

T-type Ca^2+^ channels, Cav3.1- and Cav3.2-channel, are reportedly down-regulated by chronic hypoxia in rat neonatal cardiomyocytes [12]. To confirm this in the short hypoxic period for 1–3 h, and to elucidate the mechanisms underlying hypoxia-mediated signals for the regulation of the T-type Ca^2+^ channels in cardiomyocytes in association with action of BDNF, we first examined the effect of hypoxia (1% O_2_) on the expression of the T-type Ca^2+^ channel isoforms, *Cav3.1* and *Cav3.2*, by quantifying transcript amount (Figure 1). Unexpectedly, *Cav3.1*- and *Cav3.2*-mRNA levels were prominently upregulated by an acute hypoxic insult, where the elevation was observed as early as 1 h after the exposure. At the time of hypoxia for 1 h, *Cav3.1*-mRNA and *Cav3.2*-mRNA were increased by 125% and 49%, respectively. At the time of hypoxia for 3 h, *Cav3.1*-mRNA and *Cav3.2*-mRNA were increased by 166% and 67%, respectively. Although BDNF is highly expressed in the brain, growing evidence indicates that BDNF is also expressed in the heart and is involved in cardiac pathophysiology [15,16]. We then examined the possible involvement of BDNF and its high affinity receptor tropomyosin-related kinase B (TrkB) in the hypoxic conditions of cardiomyocytes regarding the upregulation of the Cav3 channels. As we expected, expression of *Bdnf* in cardiomyocytes was significantly elevated in response to the decrease of O_2_ concentration in the cell incubator (Figure 1C). The increase of *Bdnf*-mRNA was observed as early as 1 h after hypoxic insult, which was nearly the same time course as the *Cav3*-mRNA elevation was observed. *Bdnf*-mRNA was increased by 30% at the time of hypoxia for 1 h, and by 79% at the time of hypoxia for 3 h. In contrast, the expression of *TrkB*-mRNA was unchanged by hypoxic insult during the observation period for 3 h (Figure 1D).

### 2.2. HIF-1α as a Modulator of the Cav3 Channels

**Figure 2:**
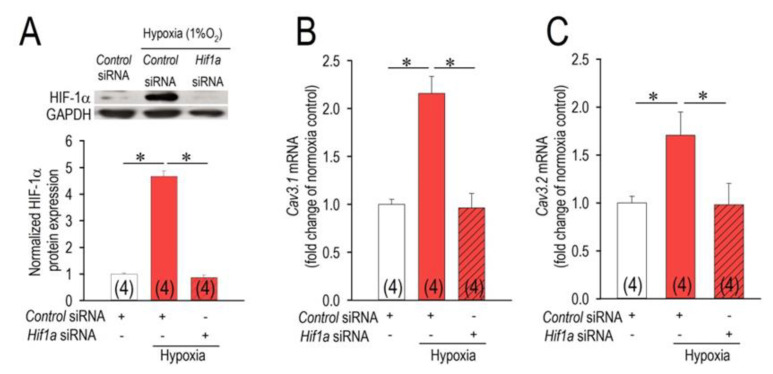
HIF-1α-dependent regulation of the *Cav3* channels in hypoxia. (**A**) Changes of HIF-1α proteins expression in cardiomyocytes exposed to normoxia or hypoxia for 3 h, and actions of *Hif1a* knockdown on them by use of control-siRNA (scramble siRNA, 10 nM) or *Hif1a*-siRNA (10 nM) transfected with Lipofectamine RNAiMAX for 48 h. After transfection, cardiomyocytes were exposed to hypoxia for 3 h, and then assessed by Western blot analysis. Representative protein images are shown above. Similar results were obtained from three independent experiments (See Figures S1 and S2). Amounts of HIF-1α proteins were normalized to those with control-siRNA in normoxia as assigned as 1.0. (**B**,**C**) Suppression of *Cav3.1*- and *Cav3.2*-mRNA by *Hif1a*-siRNA (10 nM). Experimental protocols were identical to those in panel (**A**). * *p* < 0.05, compared with those in the normoxic control group with control-siRNA or hypoxic group with *Hif1a*-siRNA as indicated. Numbers of experiments are given in parentheses (n = 4).

**Figure 3:**
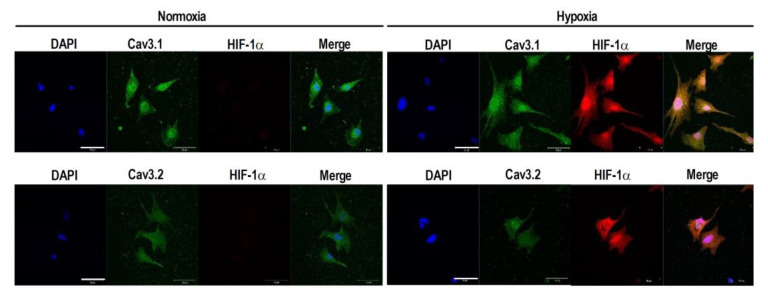
Immunocytochemistry staining for the detection of the Cav3 channels and the effect of hypoxic insult in cardiomyocytes. Cardiomyocytes were exposed to normoxia (21 % O_2_) or hypoxia (1% O_2_) for 3 h. Expression of Cav3.1 (green), Cav3.2 (green), and HIF-1α (Red) are presented. Nuclei were counterstained with DAPI (blue). Scale bar = 20 μm.

The hypoxia-inducible factors (HIFs) are the master regulators of hypoxia-driven gene expression. Because hypoxic adaptation largely depends on a family of HIFs, namely HIF-1α in the heart [20], we investigated the possible role of HIF-1α on the expression of the Cav3 channels. In our experiments of hypoxic conditions (1% O_2_) for 3 h, expression HIF-1α protein was increased more than 4 times (Figure 2A). Because the interference ability of *Hif1a*-siRNA on HIF-1α was clearly confirmed (Figure 2A), we applied the same siRNA to explore the role on the expression of *Cav3.1* and *Cav3.2*. Strikingly, the knockdown of HIF-1α by siRNA completely inhibited the upregulation of both *Cav3.1*- and *Cav3.2*-mRNA by hypoxic insult (Figure 2B,C). To visually demonstrate and to understand the distribution of HIF-1α in cardiomyocytes under the condition of hypoxia, analysis of immunocytochemical assays using antibodies targeting *Hif1a* genes were examined in combined with those of the Cav3 channels (Figure 3). The distribution of Cav3.1- and Cav3.2-Ca^2+^ channel proteins in cardiomyocytes in the hypoxic conditions represents nearly completely overlapping patterns of HIF-1α, suggesting a possible functional interaction between HIF-1α and the Cav3 channels. Importantly, the upregulation of not only *Cav3*-mRNAs but also Cav3-proteins by hypoxia was evidently confirmed by this assay.

### 2.3. BDNF Upregulates the Cav3 Channels in Hypoxia

**Figure 4:**
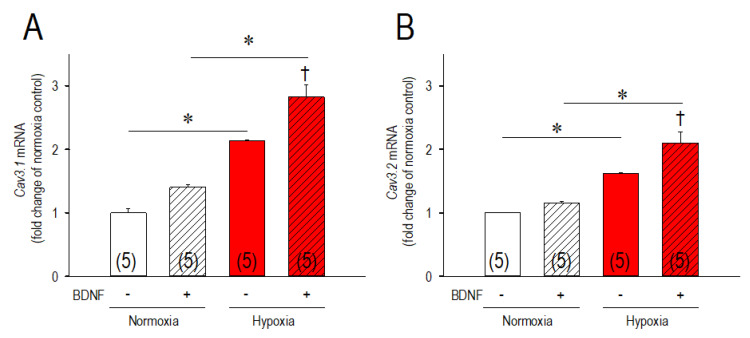
Actions of BDNF on the expression of the Cav3 channels in normoxia and hypoxia. Changes of *Cav3.1*-mRNA (**A**) and *Cav3.2*-mRNA expression (**B**) in cardiomyocytes exposed to normoxia or hypoxia for 3 h, and actions of BDNF. Recombinant BDNF protein (100 ng/mL) was applied at the time exposed to normoxia or hypoxia. Amount of each mRNA was normalized to those in normoxia (0 h) without BDNF (BDNF (−)) as assigned as 1.0. * *p* < 0.05, compared with those as indicated. ^†^ *p* < 0.05 vs. hypoxia without BDNF (BDNF (−)). Numbers of experiments are given in parentheses.

**Figure 5:**
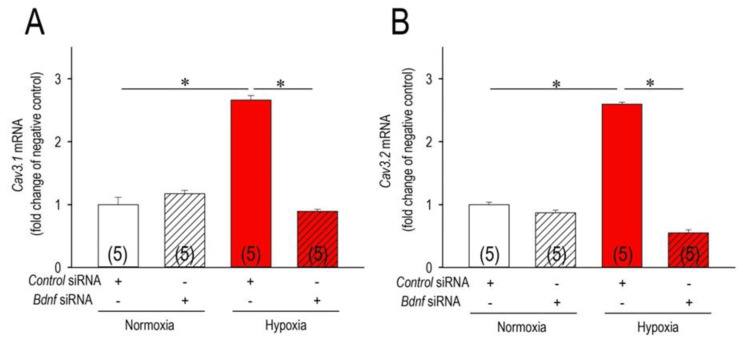
Actions of *Bdnf* knockout on the expression of *Cav3* channels in normoxia and hypoxia. Changes of Cav3.1-mRNA (**A**) and *Cav3.2*-mRNA expression (**B**) in cardiomyocytes exposed to normoxia or hypoxia for 3 h, and actions of *Bdnf* knockout by use of control-siRNA (scramble siRNA, 10 nM) or *Bdnf*-siRNA (10 nM) transfected with Lipofectamine RNAiMAX for 48 h. After transfection, cardiomyocytes were exposed to normoxia or hypoxia for 3 h, and then assessed by real-time PCR. * *p* < 0.05, compared with those as indicated. Numbers of experiments are given in parentheses (n=5).

**Figure 6:**
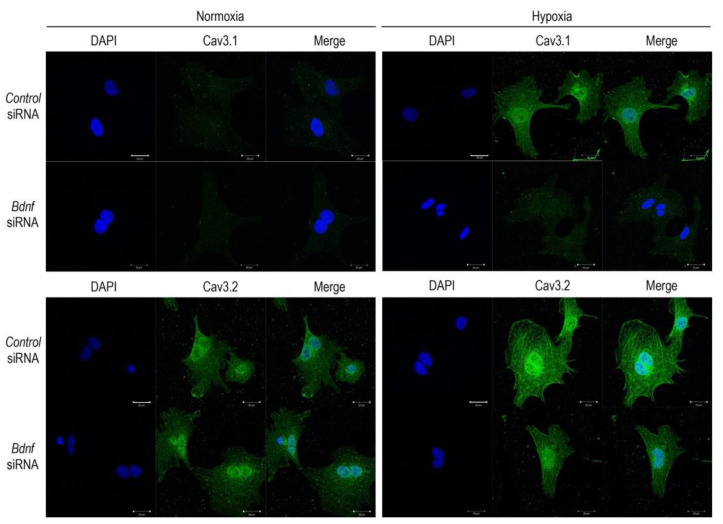
Immunocytochemistry staining for the detection of the Cav3 channels and the effect of hypoxic insult with *Bdnf* silencing in cardiomyocytes. Expression of Cav3.1 and Cav3.2 (green) are presented. Nuclei were counterstained with DAPI (blue). Actions of *Bdnf* knockdown were assessed by use of control-siRNA (scramble siRNA, 10 nM) or *bdnf*-siRNA (10 nM) transfected with Lipofectamine RNAiMAX for 48 h. After transfection, cardiomyocytes were exposed to normoxia (21% O_2_) or hypoxia (1% O_2_) for 3 h. Scale bar = 20 μm.

Because altered BDNF expression occurs under hypoxia in various cells, tissues, and organs, we intended to explore the role of BDNF on the Cav3 channels regulation in the hypoxic conditions. In this context, we incubated cardiomyocytes with or without BDNF at the conditions of normoxia/hypoxia (Figure 4). Of note, BDNF had a negligible effect on the *Cav3* channels expression at the normoxic conditions; non-significant elevation of *Cav3.1* by 41% and *Cav3.2* by 15%. However, BDNF significantly increased the expression of *Cav3.1*-mRNA and *Cav3.2*-mRNA when cardiomyocytes were incubated in the hypoxic conditions. In this experimental condition, hypoxia increased *Cav3.1*-mRNA by 114% without BDNF, and 183% with BDNF. As for *Cav3.2*, hypoxia increased *Cav3.2*-mRNA by 62% without BDNF, and 110% with BDNF. Because this effect of BDNF on *Cav3*-mRNAs was additive to the effect of hypoxia, a cooperative role of these two signaling pathways in the regulation of the Cav3 channels are suggested. To confirm the cooperative actions of BDNP to the hypoxic insult on the Cav3 channels, we then studied the knockdown effect of BDNF on the Cav3.1 and Cav3.2 channel expression (Figure 5). *Bdnf*-siRNA did not affect the expression of *Cav3.1*- and *Cav3.2*-mRNA in the normoxic conditions, which partially reconfirmed the findings of Figure 4 demonstrating a negligible effect of BDNF on the *Cav3* channels in the normoxic conditions. More importantly, *Bdnf*-siRNA completely abolished the upregulation of both *Cav3.1*- and *Cav3.2*-mRNA caused by hypoxia. These results indicate that signals for upregulations of the Cav3-channels caused by hypoxic insult are based on the signal pathways associated with those of BDNF in cardiomyocytes. To further confirm the actions of BDNF-associated pathways on the Cav3 channel proteins, results were validated by immunocytochemical assays (Figure 6). Consistent with the results in Figure 5, knockdown of BDNF or *Bdnf*-siRNA was nearly without effect on the expression of the Cav3.1 and the Cav3.2 channel in the normoxic conditions. However, in the hypoxic conditions, *Bdnf*-siRNA markedly reduced expression of both Cav3 channel proteins. These results additionally verified our notion that BDNF acts to upregulate Cav3-T-type Ca^2+^ channels in the acute hypoxic conditions in association with HIF-1α in cardiomyocytes.

## 3. Discussion

The present study demonstrates a novel BDNF-dependent upregulatory mechanism that alters the expression of the Cav3 Ca^2+^ channels in cardiomyocytes. Although pathological actions of BDNF are recognized in the heart, this investigation provides a novel aspect of BDNF actions on the regulation of cardiac excitability. This study is summar ized as follows: (1) We investigated the role of BDNF in rat neonatal cardiomyocytes exposed to normoxia (21% O_2_) and acute hypoxia (1% O_2_) in vitro for up to 3 h; (2) The expression of *Cav3.1*-, *Cav3.2*- and *Bdnf*-mRNA was upregulated by acute hypoxia; (3) BDNF upregulates *Cav3.1*- and *Ca3.2*-mRNA at the hypoxic conditions but not at the normoxic conditions; and (4) The upregulation of the Cav3 channels caused by a hypoxic insult was completely blunted by *Hif1a-*siRNA or *Bdnf*-siRNA. These findings suggest a novel action of BDNF on the T-type Ca^2+^ channels in the hypoxic conditions of the heart, which could be beneficial for cardiac function particularly in the ischemia-related conditions of the heart.

### 3.1. Acute Hypoxia Causes Upregulation of Cav3 Channels

Various studies have been performed to explore the impact of hypoxic insults on the T-type Ca^2+^ channels by use of various cell types, however, results are highly controversial [8,9,10,11,12,13]. According to studies on chromaffin cells and smooth muscle cells, chronic hypoxia caused the upregulation of Cav3.2- but not Cav3.1-T-type Ca^2+^ channel [8,9]. In the recombinant HEK-293 cells expressing the Cav3.2 channel, an acute hypoxia down-regulated the expression of the channel [10], whereas the chronic hypoxia upregulated the channel [11]. In experiments by use of rat neonatal cardiomyocytes, chronic hypoxia reduced T-type Ca^2+^ channel current accompanied by reductions of Cav3.1- and Cav3.2-mRNA [12]. In contrast, in experiments by use of adult ventricular cardiomyocytes, chronic hypoxia induced the upregulation of Cav3.2-mRNA, whereas Cav3.1- mRNA was not altered [13]. The reason for these discrepancies is unknown but may in part be related to the duration of hypoxic conditions. In this study, we clearly demonstrate that Cav3.1- and Cav3.2-mRNA were significantly upregulated by an acute hypoxic insult for 1–3 h in rat neonatal cardiomyocytes. The results were also confirmed by immunocytochemistry analysis (Figure 3 and Figure 6). Because hypoxia modulates protein expression through HIF-dependent and -independent mechanism in many cell types associated with the duration and the intensity of hypoxia [21,22], it is plausible that the short- and long-duration of hypoxia exert distinct actions on protein expressions with distinct contributions of HIF activity. Since knock-down of Hif1a completely abolished the actions of acute hypoxia on Cav3.1 and Cav3.2 in this study (Figure 2B,C), we would like to conclude that acute hypoxia up to 3 h induces upregulation of the Cav3-Ca^2+^ channels dependently on the HIF-1α actions. Obviously, we need further studies to clarify the impact of the changes in duration and intensity of hypoxia on the T-type Ca^2+^ channels.

### 3.2. HIF-1α/BDNF Involvement in Cav3 Channel Upregulation

**Figure 7:**
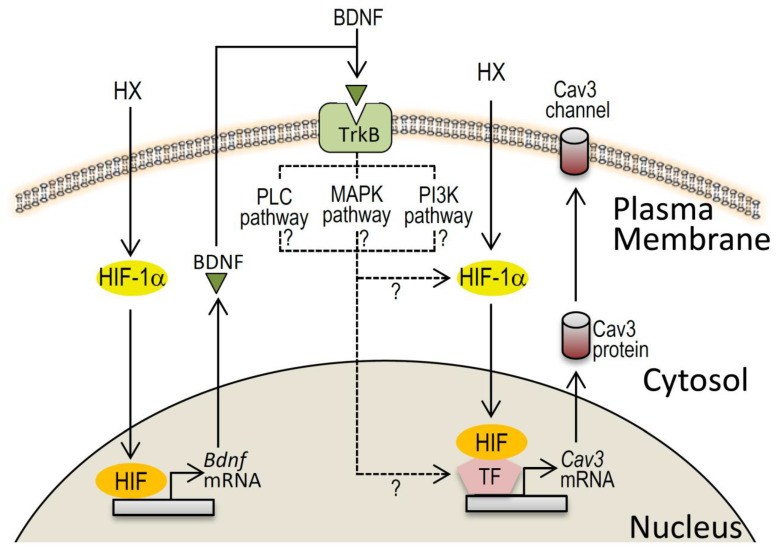
Schematic diagram of hypothetical BDNF-hypoxia interaction in regulation of the Cav3 channels in cardiomyocytes. Hypoxia (HX)/BDNF axis may work as a positive regulator for the Cav3-T-type Ca^2+^ channels through the activation of HIF-1α in cardiomyocytes, although the downstream signals of BDNF/TrkB and the targeted transcription factor(s) (TF) are unknown.

While growing evidence suggests the importance of BDNF/TrkB signals in the heart, less is known about the actions of BDNF on cardiac electrophysiological properties. It was reported that serum BDNF level was not related to the occurrence of atrial fibrillation [23]. Also, a study reported that the deletion of BDNF/TrkB signals in cardiomyocytes caused deranged Ca^2+^ homeostasis, which suggests a role of BDNF on the Ca^2+^ channel [19]. However, the roles of BDNF on cardiac Ca^2+^ channels are largely unknown. In this regard, our novel but limited findings of the upregulation of the Cav3-T-type Ca^2+^ channels in hypoxia-mediated conditions, and their modulation by BDNF may be particularly relevant in pathological aspects of BDNF-mediated cardiac excitation regulation (Figure 7). In neurons, several research works indicate that activation of BDNF-mediated pathway upregulates HIF-1α even under normoxic conditions [24,25]. Because BDNF appreciably increased *Cav3* mRNAs in normoxic conditions (Figure 4), BDNF-dependent upregulation of HIF-1α could be postulated in cardiomyocytes as well, although HIF-1α-dependent increase of BDNF signals primarily accounts for the Cav3 modulations in this study (Figure 7). Nevertheless, we were unable to identify the signal molecule(s) linking BDNF to the Cav3 channels. This is mainly because the transcription regulation pathways of *Cav3* channels genes are still mostly unknown. BDNF and its downstream pathways have profound effects on multiple functions in neurons [26].

Upon binding to the TrkB receptor, BDNF initiates intracellular signaling cascades including the phosphatidylinositol-3-kinase (PI3K) pathway, the phospholipase C (PLC) pathway, and the mitogen-activated protein kinase (MAPK) pathway. The signal molecule(s) derived from these pathways could be assumed to upregulate the Cav3 channels in association with HIFs (Figure 7). Although we have successfully demonstrated that enhanced BDNF-signaling following acute hypoxia facilitates HIF-1α-dependent upreg ulation of Cav3-T-type Ca^2+^ channels, further investigation is needed to clarify the role of these signals including the master transcription factor(s) governing the expression of the Cav3 channels.

### 3.3. Pathophysiological Significance of BDNF in the Heart Rhythm

Despite emerging evidence about the role of BDNF in the heart, there is insufficient information about its function on myocardial excitation, particularly on the heart rhythm. In some clinical and experimental investigations, controversial results have been reported in regard to the effect of BDNF on heart rate [27,28,29,30].

The peripheral application of BDNF increased heart rate in patients with amyotrophic lateral sclerosis [27], and intracerebroventricular administration of BDNF increased heart rate in mice [28]. In contrast, heart rate was elevated in *Bdnf*^+/−^ mice, which was reduced by intracerebro-ventricular administration of BDNF [29], and the application of BDNF reduced cardiomyocyte beat rate in vitro [30]. Unfortunately, we could not provide enough data to illustrate these two contrary views. Because several classes of ion channels including the T-type Ca^2+^ channels, most are the Cav3.1 and less are the Cav3.2 but not the Cav3.3 [6], contribute to pacemaker activity in the heart [31,32,33], the upregulation of Cav3-T-type Ca^2+^ channels by BDNF lend support to the view that BDNF contributes to cardiac pacing activities. Interestingly, BDNF increases T-type Ca^2+^ channel current as a short-term effect in sensory neurons via PI3K pathway [34], whereas the T-type Ca^2+^ channel was not stimulated by BDNF in chick nodose neurons [35,36]. These highly controversial results indicate that BDNF and its downstream signals are variable, possibly depending on cell types, cell cycle, environmental conditions, and others. Autonomic nerve control for heart rate may also be regulated by BDNF. Further studies are needed to reveal the complete molecular mechanism for heart rate modulation by BDNF.

In conclusion, our observations indicate a novel pathophysiological action of BDNF in association with HIF-1α on the Cav3-T-type Ca^2+^ channel expression. These results support the proposition that BDNF could be a pivotal cardiac neurotrophin for the maintenance of electrical rhythm, particularly in the diseased conditions of the heart including hypoxia/ischemia.

## 4. Materials and Methods

All experimental protocols were approved in advance by the Ethics Review Committee for Animal Experimentation of Oita University School of Medicine (No. C004003, No. G004006), and were carried out according to the guidelines for animal research of the Physiological Society of Japan to minimize the number of animals used as well as their suffering.

### 4.1. Regents

The reagents were obtained from Sigma Aldrich (St. Louis, MO, USA) or WAKO (Osaka, Japan) unless otherwise indicated. Triton X-100 was purchased from MP Biomedicals (Aurora, OH, USA). Fetal bovine serum was obtained from Biosera (Biosera, Nuaillé, Chile). Collagenase type IV was purchased from Worthington (Lakewood, NJ, USA). BDNF recombinant proteins were from Life Technologies Inc. (Gaithersburg, MD, USA). ProLong Diamond Antifade Mountant (Thermo Fisher Scientific, Waltham, MA, USA) with 4′,6-diamidino-2-phenylindole dihydrochloride (DAPI) and Alexa Fluor 488, 594-conjugated second antibody were from Molecular Probes (Eugene, OR, USA). Anti-α1G (Cav3.1) and α1H (Cav3.2) antibodies were from Santa Cruz (Santa Cruz, CA, USA). All reagents from commercial sources were of analytical grade.

### 4.2. Preparation of Neonatal Rat Cardiomyocytes and Hypoxia Treatment

All animal experiments confirmed to the Guidelines for the Care and Use of Animals at Oita University, and the NIH guidelines were approved by the institutional committee. Neonatal rat cardiomyocytes were enzymatically isolated and cultured as previously described [37,38,39]. After 24 h of culture, >70% of the cells adhered to the substrates and started to exhibit spontaneous beating. The cells were either kept under normoxia (21% O_2_) or exposed to hypoxia (1% O_2_) in incubators (Model 3130, Thermo Electron Co., Waltham, MA, USA) that maintained a constant environment (5% CO_2_ balanced with N_2_) for indicated periods of time.

### 4.3. Transfection

The cells were transfected with small interfering RNA (siRNA) using Lipofectamine RNAiMAX (Invitrogen, Carlsbad, CA, USA) in accordance with the manufacturer’s instructions. The following siRNAs were used: *Hif1a* siRNA (OriGene Technologies Inc., Rockville, MD, USA), *Bdnf* siRNA (OriGene Technologies Inc. Rockville, MD, USA), and scramble control siRNA (Santa Cruz Biotechnology, Santa Cruz, CA, USA).

### 4.4. Quantitative Real-Time PCR

The total RNA was extracted from the cardiomyocytes using TRIzol reagent (Life Technologies Inc., Carlsbad, CA, USA). Single-stranded cDNA was synthesized from 500 ng of total RNA using a Transcriptor First Strand cDNA Synthesis Kit (Roche Molecular System Inc., Alameda, CA, USA). Real-time PCR was performed on a Light cycler 480 real time PCR system (Roche, Basel, Switzerland) using SYBR Green Master Mix (Takara Bio Inc., Shiga, Japan). The primers for rat *Bdnf* (GeneBank accession no. M61175, 5′-AGCGCGAATGTGTTAGTGGT-3′ and 5′-GCAATTGTTGCCTCTTTTCT-3′), and rat neural receptor protein-tyrosine kinase (*TrkB*; GeneBank accession no. M55291, 5′-AGCAGCCCTGGTATCAGCTA-3′, 5′-TCGCCAAGTTCTGAAGGAGT-3′) were designed according to the published gene sequences. The sequences of the T-type Ca^2+^ channels (*Cav3.1* and *Cav3.2*) and internal control Glyceraldehyde-3-phosphate dehydrogenase (*Gapdf*) oligonucleotide primers used in the real-time PCR reactions are described our previous reports [32]. Data were calculated by 2^−ΔΔ^CT and presented as fold change in transcripts for each ion channels genes (*Cav3.1*, *Cav3.2*) and neurotrophic factor genes (*Bdnf*, *TrkB*) and normalized to *Gapdh* (defined as 1.0-fold).

### 4.5. Western Blot Analysis

The cells were homogenized in standard lysis buffer using protease inhibitors. Lysates were separated by using sodium dodecyl sulfate polyacrylamide gel electrophoresis (SDS-PAGE) on 10% polyacrylamide gels and transferred to polyvinylidene difluoride membranes by electroelution. Membranes were blocked in 20 mmol/L of Tris-buffered saline (TBS) solution with 1% Tween containing 5% skim milk and incubated with primary antibodies overnight at 4 °C. Polyclonal antibodies sensitive to the proteins of HIF-1α were obtained from Abcam (1:500, #ab216842, Abcam, Cambridge, MA, USA), and antibody against GAPDH was from Santa Cruz Biotechnology (Santa Cruz, CA, USA) (1:1000). Protein density was visualized on plasma membranes using horseradish peroxidase (HRP)-conjugated secondary antibodies (1:2000, American Qualex, San Clemente, CA, USA) and visualized using the enhanced chemiluminescence reagent (GE Healthcare; Waukesha, WI, USA), according to the manufacturer’s directions, and the results were exposed on Biomax Light film (Eastman Kodak; Rochester, NY, USA). Relative densitometry was determined using a computerized ImageJ software (Wayne Rasband, National Institutes of Health, Bethesda, MD, USA).

### 4.6. Immunocytochemistry

The cells were plated onto 2-well chamber slide for 24 h after knockdown of BDNF. After 3 h in culture exposed to hypoxia, cells were then rinsed in phosphate-buffered saline (PBS), fixed with 4% paraformaldehyde (5 min) and permeabilized with 0.2% Triton-X (5 min) at room temperature. We used T-type calcium channel Cav3.1 (α1G), Cav3.2 (α1H) antibodies (1:50, Santa Cruz), and HIF-1α antibody (1:50, abcam), as a secondary antibody, Alexa fluor^®^ 488, 594-conjugated donkey anti-goat IgG (H + L) antibody (1:200; Life Technologies Inc.). All antibody incubation steps were performed at room temperature and intermitted by three washing steps with 0.1% Triton-PBS. Finally, the cells were covered with ProLong Diamond Antifade Mountant with DAPI and stored at 4 °C until microscopical analysis.

### 4.7. Confocal Fluorescence Microscopy

Images were acquired using a 63× oil objective (Plan-Apochromat 63× [numerical aperture, 1.46] oil immersion objective for differential interference contrast [DIC]; Carl Zeiss, Jena, Germany). All sections were analyzed using a confocal laser microscopy system and software (LSM710, Carl Zeiss, Jena, Germany) that was built around an inverted microscope (Axio Observer Z1, Carl Zeiss, Jena, Germany) as previously described [40]. The images were saved in TIFF format and analyzed by ImageJ software (Wayne Rasband, National Institutes of Health, Bethesda, MD, USA).

### 4.8. Statistics

The experiments were performed at least three times. Statistical analysis was conducted using SigmaPlot 14.0 (SigmaPlot version 14.0 -Systat Software, Inc., London, UK). Values are mean ± S.E. Values of *p* were calculated using unpaired Student’s t-test for differences between two groups, and one-way ANOVA for differences among multiple groups. Values of *p* < 0.05 were considered statistically significant.

## Data Availability

The data in this study are available from the corresponding author on reasonable request.

## Supplementary Materials

The following are available online at https://www.mdpi.com/article/10.3390/membranes11070470/s1, Figure S1: The raw WB images, Figure S2: HIF-1α and GAPDH images.

## Abbreviations

BDNF	Brain-derived neurotrophic factor
HIF-1a	hypoxia-inducible factor 1a
TrkB	tropomyosin-related kinase receptor B
siRNA	small interfering RNA
DAPI	4′,6-diamidino-2-phenylindole dihydrochloride
HX	hypoxia
PLC	phospholipase C
MAPK	mitogen-activated protein kinase
PI3K	phosphatidylinositol-3-kinase
