# Supplementary material for: Enhanced BDNF Actions Following Acute Hypoxia Facilitate HIF-1α-Dependent Upregulation of Cav3-T-Type Ca2+ Channels in Rat Cardiomyocytes
Source: Membranes (Basel). 2021 Jun 25;11(7):470. doi: 10.3390/membranes11070470 (PMC8307968; doi:10.3390/membranes11070470)
Supplement: Supplementary file 1 [file membranes-11-00470-s001.zip › membranes-1271258-supplementary.pdf]

membranes

*Supplementary Material*

# Enhanced BDNF Actions Following Acute Hypoxia Facilitate HIF-1α-Dependent Upregulation of Cav3-T-type $Ca^{2+}$ Channels in Rat Cardiomyocytes

**Masaki Morishima[1, 2], Takafumi Fujita[2], Satoshi Osagawa[2], Hiroshi Kubota[2], Katsushige Ono[2,*]**

[1] Department of Food and Nutrition, Kindai University Faculty of Agriculture, Nara 631-8505, Japan; mmoris@nara.kindai.ac.jp
[2] Department of Pathophysiology, Oita University School of Medicine, Oita 879-5593, Japan; cardio2000xt@gmail.com (T.F.); r2d20012001@gmail.com (S.O.); heartland20012001@gmail.com (H.K.)
* Correspondence: ono@oita-u.ac.jp; Tel.: +81-975-865-650

## 1. Supplemental Data for western blot image in Figure 2 [Figure 2A]

HIF-1α antibody (1:500, #ab216842, Abcam) GAPDH (1:1000, Santa Cruz Biotechnology)

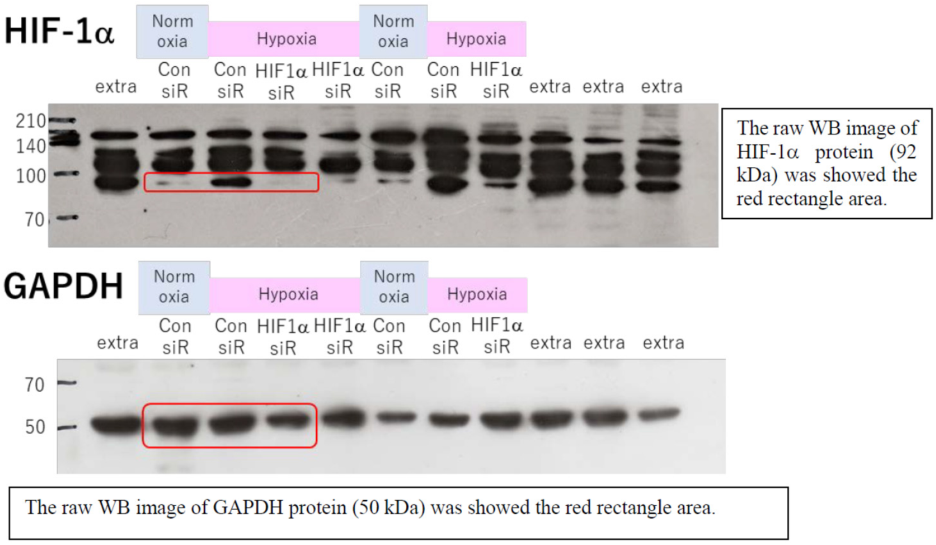


**Figure S1.** The raw WB images.

Next two experiments for HIF-1α and GAPDH images in below panel were not used in the manuscript, although they show the nearly identical results.

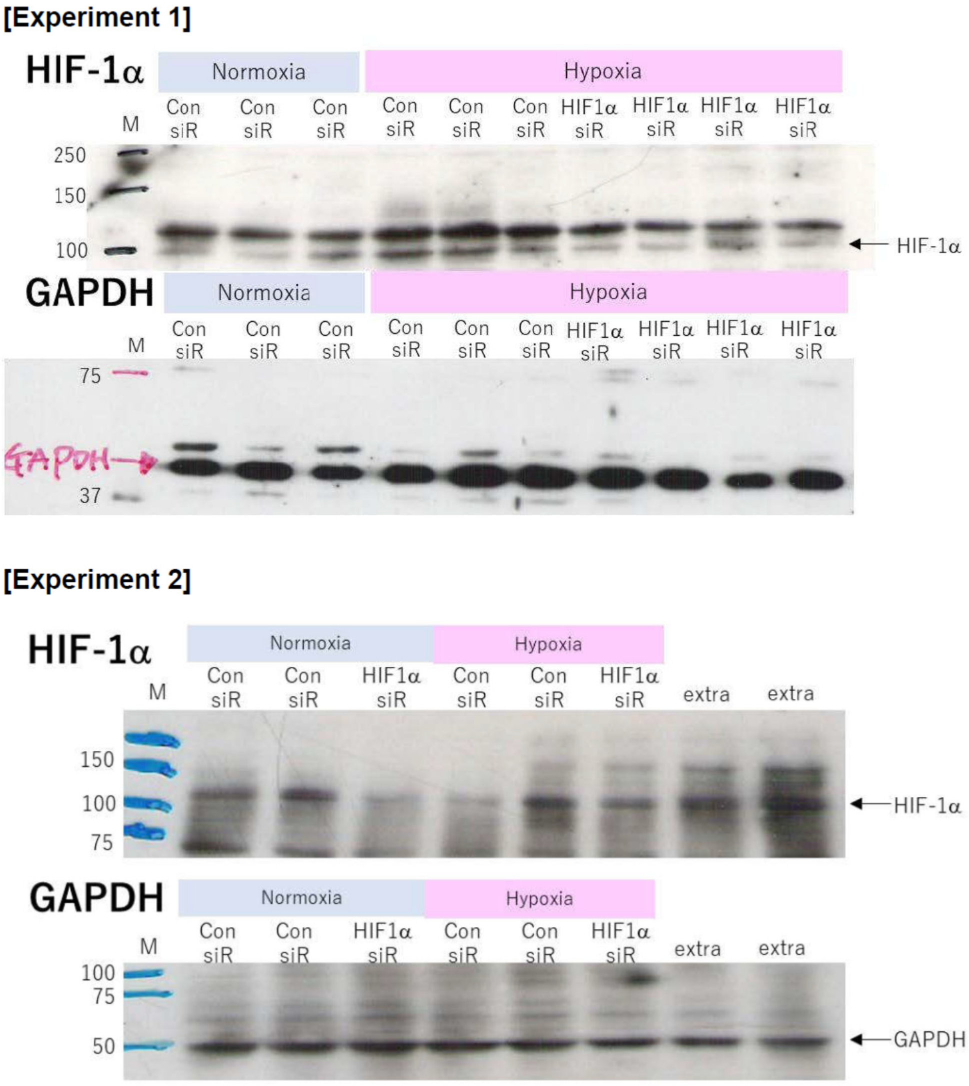


**Figure S2.** HIF-1α and GAPDH images.

Cytoplasmic proteins from cardiomyocytes were prepared using RIPA lysis buffer. Samples containing 40 μg were denatured at 95°C for 5 min in loading buffer [Tris -HCl (pH6.8) 250 mM, 4% SDS, 1% β-mercaptoethanol, 1% bromophenol blue, and 20% glycerol], separated by SDS -polyacrylamide gel electrophoresis using 10% polyacrylamide gel, and then transferred from the gel to a PVDF membrane (Hybond-P; GE Healthcare Bio-Sciences, Piscataway, NJ, USA). The blot was visualized with anti -rabbit IgG horseradish peroxidase-conjugated secondary antibodies (1:2000, American Qualex, CA, USA) and an ECL prime Western Blotting Detection System (GE Healthcare Bio-Sciences Piscataway, NJ, USA) and the results were exposed on Biomax Light film (Eastman Kodak; Rochester, NY, USA). Because we can see some non-specific band on the membranes, we tried to abolish by modulating a blocking time or altering a dilution rate of the secondary antibody. However, we could not absolutely remove the non-specific band, so we assessed target proteins by using visualized protein marker as an indication. To eliminate "smiling band" of protein in SDS-PAGE, we used another protein samples such as an "extra" in this study.
